# Roles and Mechanisms of Obstructive Sleep Apnea-Hypopnea Syndrome and Chronic Intermittent Hypoxia in Atherosclerosis: Evidence and Prospective

**DOI:** 10.1155/2016/8215082

**Published:** 2016-05-16

**Authors:** Linqin Ma, Jingchun Zhang, Yue Liu

**Affiliations:** ^1^Cardiovascular Diseases Centre, Xiyuan Hospital of China Academy of Chinese Medical Sciences, Beijing 100091, China; ^2^China Heart Institute of Chinese Medicine, China Academy of Chinese Medical Sciences, Beijing 100091, China

## Abstract

The morbidity and mortality of obstructive sleep apnea-hypopnea syndrome (OSAHS) are regarded as consequences of its adverse effects on the cardiovascular system. Chronic intermittent hypoxia (CIH) induced by OSAHS can result in vascular endothelial injury, thus promoting development of atherosclerosis (AS). Studies have shown that CIH is an independent risk factor for the occurrence and development of AS, but the underlying mechanism remains unclear. Here, we review clinical and fundamental studies reported during the last 10 years on the occurrence and development of AS mediated by CIH, focusing on inflammation, oxidative stress, insulin resistance, cell apoptosis, vascular endothelial injury, platelet activation, and neuroendocrine disorders. This review will offer current evidence and perspective to researchers for the development of effective intervention strategies for OSAHS-related cardiocerebrovascular diseases.

## 1. Introduction

Obstructive sleep apnea-hypopnea syndrome (OSAHS) is a common disease worldwide. A large-scale meta-analysis [1] has shown that approximately 1 in 5 adults suffer from moderate OSAHS, while 1 in 15 adults suffer from severe OSAHS. OSAHS can induce a great amount of damage to all body systems and is associated with increased secondary cardiovascular morbidity and mortality. OSAHS is also an independent risk factor for cardiovascular disease [2–4], resulting in hypertension, stroke, myocardial infarction (MI), cardiac failure, arrhythmia [5], and sudden death during the night [6]. These conditions are often the underlying causes of disability and death. In the past 20 years, many clinical and fundamental studies have provided evidence for a correlation between OSAHS and cardiovascular diseases. Of many associated factors, chronic intermittent hypoxia (CIH), in particular, is one of the hallmark features in OSAHS. The most common characteristic of OSAHS is a long-term, repetitive cycle of anoxia-reoxygenation during sleep, also known as CIH, and it is a major underlying culprit in OSAHS-induced cardiocerebrovascular complications. A recent study has suggested that CIH promotes vascular injury in OSAHS, which in turn leads to the occurrence of atherosclerosis (AS) [7], accelerating the development of cardiocerebrovascular diseases such as coronary heart disease and stroke.

A large number of clinical investigations [8, 9] and experimental studies [10, 11] focusing on the common risk factors, pathologic evolution, and underlying mechanisms of OSAHS have demonstrated that CIH-mediated AS plays an important role in OSAHS-related cardiocerebrovascular diseases. However, disagreements regarding the underlying mechanism among these studies remain. This review of clinical and fundamental research summarizes the various mechanisms whereby CIH promotes the occurrence and development of AS, focusing on the molecular mechanisms and signaling pathways. This review will aid in the identification of novel molecular targets for prevention and treatment strategies of OSAHS-induced AS complications.

## 2. CIH Promotes Occurrence and Development of Atherosclerosis and Related Cardiovascular Diseases

Clinicians have found that OSAHS often accompanies AS and that OSAHS increases risk of developing AS and related cardiocerebrovascular diseases. A cohort study [12] found that progress and deterioration of sleep disordered breathing over 5 years were related to the incidence of cardiovascular events. The correlation between increased apnea-hypopnea index (AHI), which is an important indicator of OSAHS severity, and incident myocardial infarction (MI) was statistically significant. After 24 months of follow-up in another retrospective analysis [13], it was found that ST-elevation myocardial infarction (STEMI) patients with OSAHS had more severe ventricular hypertrophy and poorer cardiac function. It has also been found that there is a positive correlation between AHI and coronary atherosclerotic plaque volume in OSAHS patients [14]. These findings indicate that there is a high correlation between OSAHS and coronary atherosclerotic heart disease.

OSAHS patients exposed to CIH have an increased risk of suffering from obesity, abnormal lipid metabolism, hyperglycemia, and hypertension, and these abnormities are major risk factors for the development of AS [15–18]. OSAHS and CIH can lead to occurrence and development of these risk factors, which in turn predispose for AS and related cardiovascular diseases. There is high prevalence of OSAHS in obese patients [19, 20]; these patients often have interrupted sleep, drowsiness during daytime, and decreased physical activities, which could cause increased fat mass. Leptin is an endogenous peptide hormone encoded by the* obese* (*ob*) gene. Leptin is synthesized and secreted by adipose cells and promotes lipolysis and suppresses fat synthesis. A systematic review [21] found that gene diversity in the leptin receptor is related to decreased risk for OSAHS in Europeans. OSAHS also plays an important role in abnormal lipid metabolism. In OSAHS patients, the expression of hypoxia inducible factor-1 (HIF-1) is upregulated. Activation of the HIF-1*α*/SREBP-1c/FAS pathway is a key molecular mechanism leading to development of abnormal lipid metabolism in liver cells exposed to CIH [22]. OSAHS is strongly correlated with occurrence and development of insulin resistance (IR) and diabetes. A study [23] using polysomnography (PSG) was conducted among 118 nondiabetic subjects to compare glucose tolerance and fasting insulin level between 39 non-OSAHS patients and 79 OSAHS patients and to observe the influence of OSAHS on the metabolism kinetics of glucose and insulin* in vivo*. These investigators found that, independent of obesity factors, OSAHS is strongly correlated with decreased insulin sensitivity, reduction of glucose utilization rate, and dysfunction of pancreatic *β* cells. OSAHS may therefore increase the risk for abnormal glucose tolerance and the occurrence of type 2 diabetes. OSAHS is also related to the occurrence of refractory hypertension. A cross-sectional study found that CIH plays an important role in OSAHS-related hypertension incidents [24]. Both CIH and frequent awakening at night are capable of causing elevation of blood pressure in OSAHS patients, and the study revealed an increased severity of hypertension-mediated target organ injury in these patients, compared with patients having simple hypertension. Currently, continuous positive airway pressure (CPAP) is the main treatment for OSAHS patients, in which positive pressure air is continuously applied through a mask into the airway to improve patients' oxygen status. It has been reported that CPAP treatment plays a key role in improving biomarkers of these metabolic disorders [25–28], such as total cholesterol (TC), adiponectin, HbA1c, and insulin.

Animal studies have been conducted to clarify the interrelationship between OSAHS, CIH, and AS. A study [10] investigated the effects of intermittent air and CIH on the formation of atherosclerotic plaques in ApoE^−/−^ mice, which have increased susceptibility to AS. The results indicated that inducing CIH for a 4- or 12-week period promoted formation of AS plaques in the aorta, compared with control mice. In addition, systolic blood pressure was elevated in week 4 in CIH-exposed mice, and diastolic pressure was also elevated in week 12. Other animal studies have identified additional factors that mediate the effects of OSAHS and CIH on the development of AS, such as the elevation of endogenous erythropoietin (EPO) [29] and the induction of coronary artery calcification [30, 31].

The above studies suggest that OSAHS and CIH play a significant role in controlling the incidence and development of AS. On the other hand, AS-related risk factors can accelerate the occurrence of OSAHS. Obesity, male sex, age, menopause, and smoking are also considered risk factors of OSAHS [32]. A clinical study found that type 2 diabetes is independently associated with OSAHS [33]. Patients with abnormal glycolipid metabolism are more prone to develop structural changes in the upper respiratory tract if they are obese or have metabolic syndrome (MS), and this in turn can induce airway stenosis, accelerating or exacerbating the occurrence of OSAHS. Thus, OSAHS, a variety of metabolic disorders, and AS have a few common risk factors and mechanisms. OSAHS and various metabolic disorders may have a synergistic effect on the occurrence and development of AS.

## 3. CIH-Induced Atherosclerosis: Possible Mechanisms

### 3.1. Inflammatory Response

Studies on AS [34–36] have shown that the occurrence and development of AS can be regarded as a chronic inflammatory process that involves multiple inflammatory cell types and mediators and that vascular inflammatory injury can also induce AS. Numerous studies have shown that CIH can affect the production of various inflammatory factors and cytokines. For example, Muraki et al. [37] have demonstrated that nocturnal intermittent hypoxia is directly and positively correlated with the level of high-sensitivity C-reactive protein (hs-CRP). It has been shown [38] that CIH upregulates expression and secretion of interleukin-8 (IL-8), which in turn promotes inflammation and AS; increased expression of cytokines such as E-selectin has also been found to be highly correlated with CIH exposure [39]. Collectively, these studies suggest that activation of inflammatory pathways is the primary mechanism of CIH-mediated AS.

Nuclear factor *κ*B (NF-*κ*B) is one of the most extensively studied inflammatory factors and is considered to be the key regulator in various inflammatory responses. Inflammatory factors such as tumor necrosis factor-*α* (TNF-*α*), IL-6, IL-8, and intercellular cell adhesion molecule-1 (ICAM-1) can initiate inflammatory responses via induction of NF-*κ*B expression. CIH-induced inflammatory responses in various tissues and cells can also mediate the occurrence of downstream biological effects by regulating the NF-*κ*B pathway. The heterodimeric NF-*κ*B (p50-p65) in the cytoplasm is biologically inactive when bound by the inhibitor I*κ*B. When cells are stimulated by external factors (such as viruses, ultraviolet rays, cytokines, and oxidative stress), I*κ*B is phosphorylated, allowing the activated NF-*κ*B to enter the nucleus and bind to enhancer elements to initiate various inflammatory responses. Researchers [11] have compared wild-type and NF-*κ*B p50 knockout C57BL/6 mice subjected to a normal or high-fat diet, intermittent air, or CIH, respectively, to investigate their effects on the formation of aortic AS plaques. The results indicated that, in wild-type mice, high-fat diet did not induce significant AS changes, while 20 weeks after CIH induction and high-fat diet significant AS plaque formation was observed. On the other hand, in p50 knockout mice, NF-*κ*B activation and AS plaque formation triggered by CIH and high-fat diet treatment were significantly suppressed. The elevation of total cholesterol (TC) and formation of foam cells were also reduced, indicating that knockout of the p50 gene inhibited inflammation in the vascular wall. This study demonstrated that inhibition of NF-*κ*B activation can decrease the severity of AS caused by CIH and high-fat diet and that NF-*κ*B may be the common path and core mechanism for the occurrence of AS following CIH and high-fat diet. Another study also showed a clear relationship between CIH and AS. By observing C57BL/6 mice exposed to 14 or 35 days of intermittent hypoxia, it was found that leukocyte rolling and adhesion molecule ICAM-1 expression were enhanced in mesenteric resistance vessels and with NF-*κ*B activation [40].

Another study [41] that used intermittent hypoxia/reoxygenation- (IHR-) exposed human umbilical vein hybridoma cells (EA.hy926) found that inhibition of NF-*κ*B significantly improved the IH-mediated upregulation of inflammatory factors such as IL-6 and IL-8 after 64 cycles of IHR. This study also indicated that NF-*κ*B activation may be one of the mechanisms whereby IH induces vascular inflammatory lesions or even AS. In addition, the upregulation of inflammatory cytokines like IL-6 and monocyte chemoattractant protein-1 in the endothelial cells under intermittent hypoxia exposure conditions provides more direct evidence for vascular endothelial damage and the related AS induced by IH [42].

Numerous studies on the upstream signaling pathway of NF-*κ*B have suggested that, in addition to the common upstream molecules such as TNF-*α* and IL-6, p38 MAPK is also a key therapeutic target. IHR has been shown to activate the p38 MAPK/NF-*κ*B pathway when cattle arterial endothelial cells were stimulated with IHR* in vitro* [43]. Specific inhibition of p38 MAPK by SB 203580 caused a significant reduction in IHR-induced NF-*κ*B activation. Activation of NF-*κ*B by IHR-mediated activation of the IKK complex and phosphorylation of I*κ*B-*α* was further confirmed in HeLa cells, indicating that IH can activate NF-*κ*B via p38 MAPK and thereby mediate IH-related cardiovascular inflammation.

However, an inflammatory mechanism is not likely to be the only explanation for CIH-related AS. When observing changes in expression of TNF-*α* and IL-1*β* in the carotid body of SD rats after 21 days of CIH exposure, researchers [44] found that CIH exposure significantly enhanced the levels of TNF-*α* and IL-1*β* and that ibuprofen effectively suppressed the CIH-induced enhancement of TNF-*α* and IL-1*β* expression. However, ibuprofen failed to completely eliminate the biochemical reactions of carotid body in response to hypoxia, indicating that inhibition of inflammation could not completely reverse the damaging effects of CIH. Thus, the increase in inflammatory response can only serve as a partial explanation for the mechanism underlying CIH-mediated AS formation.

### 3.2. Oxidative Stress

Oxidative stress, a result of the repetitive anoxia-reoxygenation cycle of CIH, plays a key role in OSAHS-related cardiocerebrovascular diseases [45]. Several studies have shown that OSAHS or CIH is closely linked to the upregulation of peroxidative markers in the body such as reactive oxygen species (ROS) [46], malondialdehyde (MDA) [47], and superoxide dismutase (SOD) [36]. Another study has indicated that oxidative stress is also a key factor in initiating vascular endothelial injury and AS formation [48].

The molecular mechanism and signaling pathways that mediate oxidative stress during CIH are not yet conclusively established and are being extensively studied. The process of repeated hypoxia/reoxygenation increases ROS production. ROS can function as signaling molecules and regulate some signal transduction pathways, which may lead to pathological changes. ROS targets include mitogen-activated protein kinase (MAPK), activator protein-1, sterol regulatory element binding proteins (SREBPs), GATA-4, NF-*κ*B, NOTCH-1, and paraoxonase-1 [49–51]. Metallothionein (MT) is one of the most potent proteins involved in eliminating free radicals in the body. It has strong antioxidant activity and can be used as an antioxidant. A study [52] evaluating oxidative stress, inflammatory responses, and apoptosis after 3 days, 1 week, 3 weeks, and 8 weeks of CIH exposure in MT knockout and wild-type 129S1 mice found that, compared to wild-type mice, CIH-mediated artery fibrosis, artery inflammation and oxidative damage, and apoptosis appeared earlier and were more severe in MT knockout mice. Additionally, the arterial MT level increased in 3 days (early stage) but significantly decreased in the later stage, indicating that CIH can trigger AS by inducing inflammation and oxidative stress reactions and that MT may play an important role in this process. Stimulation of the oxidative stress-induced heme oxygenase-1 (HO-1) aggravated CIH-mediated oxidative stress and apoptosis but not the release of inflammatory factors in IHR-treated EA.hy926 cells* in vitro*, indicating that CIH exposure can accelerate apoptosis by enhancing oxidative stress in endothelial cells, while the HO-1 pathway may be one of the important mechanisms for CIH-induced vascular endothelial injury [41].

Reviewing the clinical studies and fundamental research pertaining to CIH-related AS, it can be concluded that inflammation and oxidative stress are the two characteristic pathological changes in OSAHS patients and in tissues and cells exposed to CIH conditions in experimental simulations. A number of studies are being carried out to study inflammatory and oxidative stress effects of CIH and the mechanisms involved therein.

### 3.3. Insulin Resistance

Insulin resistance (IR) is one of the main pathological bases of metabolic syndrome (MS), which is the aggregation of multiple metabolic abnormalities including hyperglycemia, hyperlipidemia, hypertension, and obesity. Since OSAHS can result in significantly increased prevalence of MS [15] and there is an association between metabolic syndrome and AS [53], it is speculated that IR might be one of the essential mechanisms involved in CIH-mediated AS. Research has indicated that OSAHS can enhance vascular endothelial injury caused by abnormal glucose and lipid metabolism to promote occurrence and development of AS and can increase the risk of AS in patients with diabetes or hyperglycemia [7]. Another study [54] found that fasting blood glucose and fasting serum insulin were significantly elevated in CIH-exposed mice compared to intermittent air-exposed mice, indicating that CIH caused decreased insulin sensitivity and abnormal glucose metabolism. Moreover, increased TC and low density lipoprotein (LDL) and upregulation of liver enzymes, lipoprotein secretion, and stearoyl-CoA desaturase-1 (SCD-1, which is selectively suppressed by leptin) in mice subjected to CIH and high-fat diet compared to mice subjected to intermittent air and high-fat diet indicated that CIH can independently mediate abnormal lipid metabolism. Researchers found that CIH could increase plaque size in the aortic sinus and the descending aorta in ApoE^−/−^ mice, which was mainly due to increased serum lipids and blood pressure [10]. Additionally, significant AS injuries were found in the aortic origin and descending aorta in mice subjected to CIH and high-fat diet. Therefore, studies have demonstrated a significant correlation between CIH, IR, and AS.

To date, there have been many reports on the mechanisms and signaling pathways of the previously mentioned IR-mediated effects. A study has demonstrated [55] that, in the white adipose tissue of mice, hypoxia exposure regulated the expression of lysyl oxidase and other target genes by promoting the upregulation of HIF-1 and thus exacerbated the fibrosis of adipose tissue and inflammatory responses to induce IR. Angiopoietin-like 4 (Angptl4) is one of the essential participating factors in early stage AS pathology in MS patients and is closely related to IR. Studies [56] of the metabolic disorder induced by CIH and its effect on the expression of the HIF-1/Angptl4 pathway in ApoE^−/−^ mice after 4 weeks of CIH exposure showed that hypoxia significantly elevated Angptl4 levels in adipose tissue, inhibited lipoprotein lipase in adipose tissue, enhanced plasma TC and very low density lipoprotein-cholesterol (vLDL-C), and increased the area of AS plaques. However, these effects were inhibited by Angptl4-neutralizing antibody. In HIF-1*α* heterozygous knockout (HIF-1*α* ^+/−^) mice subjected to CIH, CIH-induced elevations of plasma TC, and adipose tissue Angptl4 were reversed, while adipose tissue HIF-1*α* overexpression in transgenic mice resulted in hyperlipidemia and an increase of Angptl4. The findings of this study suggest that this pathway may be one of the important mechanisms of CIH-mediated abnormal lipid metabolism and even AS formation. A recent study [57] has found that expression of SREBP-1 was highly upregulated in retinal white adipose tissue in patients with metabolic syndrome, indicating that SREBPs could play a vital role in CIH-mediated IR. Not only is IR one of the important pathological effects induced by CIH, but also it is a key pathogenic factor of AS. It has a vital role in the CIH-mediated AS process and has become one of the new areas of active research.

### 3.4. Cell Apoptosis

Cell apoptosis plays a role in various physiological and pathological processes such as inflammation, carcinogenesis, and aging and is also important in the mechanism of CIH-mediated AS.

A clinical study [58] has found that hypoxia in OSAHS patients is associated with decreased neutrophil apoptosis. Another study [59] using* in vitro* IH-treated human neutrophils has shown that the imbalance of Bax and Mcl-1 of the Bcl-2 family, which control cell apoptosis and survival, respectively, prolonged the survival of neutrophils. These neutrophils in turn could induce IH-mediated cardiovascular injuries such as sustained inflammatory responses and tissue damage, the underlying mechanism of which may be related to oxidizing radicals and proteolytic enzymes formed during neutrophil-endothelium interactions [58]. In addition, the ERK1/2 signaling pathway induced by IH may play an important role in regulating the balance of protein function in the Bax/Mcl-1 signaling pathway. Extracellular regulatory protein kinase (ERK) is activated via phosphorylation by various growth factors, ionizing radiation, and hydrogen peroxide. Activated ERK enters the cell nucleus to act on transcription factors, which in turn promote transcription and expression of inflammation- and apoptosis-related genes. Research has showed that ERK can serve as an important signaling pathway in promoting proliferation of vascular smooth muscle cells (VSMCs) and subsequently mediates diabetes-related AS [60]. In addition, the p38 MAPK signaling pathway is also a possible mechanism for IH-mediated AS; oxidized low density lipoprotein (ox-LDL) and natural LDL formed by oxidative modification of lipoproteins are closely associated with inflammatory responses and AS. As a component of ox-LDL, a study [61] has shown that lysophosphatidylcholine induced apoptosis of human vascular endothelial cells via the p38 MAPK signaling pathway.

Apoptosis is a complex cascade reaction. Under IH conditions, apoptosis in different parts can result in variations in the underlying mechanisms involved in the development of AS. For example, vascular endothelial cell apoptosis is the characteristic and important pathological change during the progress of CIH-induced cardiovascular diseases. It had been reported that the level of circulating apoptotic endothelial cells is higher in patients with OSAHS compared to non-OSAHS subjects [62]. CPAP treatment improves hypoxia and decreases apoptosis of endothelial cells [63]. Furthermore, insufficient apoptosis of inflammatory cells promotes the persistence of AS inflammatory responses. Pancreatic *β* cell apoptosis can also induce IR and thus mediate abnormal glycolipid metabolism to promote the occurrence of AS. Apoptosis can be the result of many pathological effects caused by CIH. It not only aggravates the AS-promoting effects such as inflammation and IR, but also serves as an important link that mediates the relationship between CIH and AS.

### 3.5. Vascular Endothelial Injury

Vascular endothelial injury is the initial step in AS formation [64] and CIH-mediated AS. Exposure of ApoE^−/−^ mice to CIH conditions for 6 weeks showed significant impairment of endothelial function compared to the intermittent air exposure group [65]. CIH resulted in endothelial cell dysfunction by enhancing ROS levels and inflammatory mechanisms; however, anti-inflammatory and antioxidant combination therapy using infliximab and glutathione inhibited CIH-induced vascular injury, indicating that CIH can exacerbate vascular endothelial injury and promote AS formation.

In addition to inflammatory mechanisms and oxidative stress injuries, imbalance of vasoactive factor expression, upregulation of intercellular adhesion molecules, and apoptosis of vascular endothelial cells are important mechanisms of CIH-mediated vascular endothelial injury. In OSAHS patients without clear cardiovascular diseases, expression of CD34 and CD31, markers of endothelial progenitor cells (EPCs), is significantly reduced, while vascular endothelial growth factor (VEGF) is markedly elevated, indicating that these vasoactive factors may be involved in the formation of vascular endothelial injuries in OSAHS patients [66]. In a study of ICAM-1 and vascular cell adhesion molecule-1 (VCAM-1) levels in the blood of OSAHS patients, multivariate logistic regression analysis showed that OSAHS was independently associated with high levels of ICAM-1 and VCAM-1 expression [67].

As opposed to the previously mentioned mechanisms of perturbed molecular and cellular levels of vasoactive factors, vascular endothelial injury is more of a macroconcept that can link the other mechanisms with pathologically visible AS as the key process. Mechanisms such as inflammatory responses, oxidative stress, and apoptosis can all initiate the formation and development of AS by mediating vascular endothelial injuries. Therefore, protection of vascular endothelium is an important strategy in treating OSAHS or CIH-related AS diseases, and prevention of vascular endothelial injury is a vital step in blocking the occurrence of cardiocerebrovascular diseases.

### 3.6. Platelet Aggregation and Activation

Platelets participate in the formation and development of AS and also promote the instability and rupture of AS plaques. Therefore, inhibition of platelet aggregation and activation can potentially prevent AS-associated secondary cardiovascular diseases [68]. OSAHS patients have abnormal platelet aggregation and activation [69]. In a clinical study of platelet activity in obese patients, those with OSAHS had a higher degree of oxygen desaturation upon platelet activation than those without OSAHS [70]. Another clinical study [71] found that the degree of platelet activation was greater in patients having moderate and severe OSAHS compared to those having mild OSAHS and that CPAP therapy significantly improved inappropriate platelet aggregation in some patients. Platelet microparticles (PMPs) are membranous microvesicles released during the process of platelet activation and are a type of blood cell-derived particle. PMPs are equipped with proinflammatory, procoagulant, and antiendothelial functions. Patients having mild OSAHS had significantly increased levels of platelet- and leukocyte-derived particles compared to controls, indicating that, despite the milder clinical symptoms in these patients, the potential for increased risk for cardiocerebrovascular disease, inappropriate platelet activation, and AS is still present [72].

Platelet activation is a complex process that involves multiple signaling pathways, such as the cyclic adenosine monophosphate-protein kinase A (cAMP-PKA), phosphatidylinositol 3-kinase (PI3K), and MAPK pathways. However, there is still a lack of evidence for a specific signal transduction mechanism of CIH-mediated platelet activation. Studying the functional characteristics of CIH-induced platelet aggregation and activation and vascular endothelial injury will help in understanding the mechanism of AS formation and may provide new insights for prevention of OSAHS-associated secondary AS vascular diseases.

### 3.7. Neuroendocrine Disorder

OSAHS patients have sustained excitation of the sympathetic nerve induced by repeated nocturnal arousals, daytime drowsiness, and hypoxia, which in turn can stimulate the release of catecholamine and the activation of renin-angiotensin-aldosterone system and thereby induce neurohumoral dysregulation. Neuroendocrine disorder factors lead to AS in a more indirect way and are significantly associated with AS-mediating factors such as fluctuation of blood pressure, glycolipid metabolic disorder, vascular endothelial injury, and platelet activation, as well as formation of artery plaques and rupture.

A prospective clinical study [73] compared muscle sympathetic nerve activity reflecting increased sympathetic outflow between MS patients with OSAHS and without OSAHS. The study found that OSAHS increased sympathetic nerve activity by increasing sympathetic peripheral and central chemoreflex response. Studies [74, 75] have shown that hypoxia-induced carotid body dysfunction is related to processes such as the upregulation of renin-angiotensin system, inflammation in the carotid body, and oxidative stress. CIH interferes with the normal function of carotid body in maintaining dynamic oxygen equilibrium in OSAHS patients and thus induces secondary damage.

## 4. Reflection: Is the Link between CIH and AS Primary or Secondary?

Based on the above, it can be seen that CIH plays an important role in promoting occurrence and development of AS. However, the underlying mechanisms that link CIH with AS are complex. A number of factors, like lipids, serum glucose and inflammatory cells, inflammatory factors, cytokines, ROS, apoptotic cells, and so on, mutually influence each other. It is still unclear whether the link between CIH and AS is primary or secondary. Generally speaking, CIH may lead to AS as a secondary effect by aggravating these risk factors. For instance, OSAHS is always accompanied by other metabolic disorders, like obesity or abnormal glucose metabolism, which are the causes of OSAHS or hypoxia as well [33, 76]. Based on research carried out till date, it is difficult to tell whether CIH or the metabolic disorder itself causes AS through a molecular mechanism or signaling pathway. The SREBP-1c/FAS signaling pathway [77] and the NF-*κ*B signaling pathway are important molecular mechanisms involved in the process of different metabolic disorders and AS. CIH plays a role in these mechanisms, instead of being a key mechanism for CIH-induced AS. When investigating and analyzing the mechanism involved in AS occurrence and development, researchers must objectively evaluate the effect of CIH on AS.

## 5. Conclusion and Prospects

As shown above, CIH has become a widely researched area in recent years. Research has shown that CIH makes it more difficult for the body to adapt and causes damage more readily than persistent hypoxia. In contrast to persistent hypoxia, CIH is characterized by intermittent oxygenation following hypoxia, which is similar to the process of ischemia-reperfusion injury, and it is an important mechanism of OSAHS target organ injury. As the most common and important sleep-related breathing disorder, OSAHS is closely associated with the development and exacerbation of cardiocerebrovascular diseases such as hypertension, coronary heart disease, and stroke [78–80]. The core pathological manifestations of OSAHS rely on the strong correlation between CIH and AS, and it is now well known that CIH can promote the occurrence and development of AS. An in-depth study of the mechanism underlying the correlation between these conditions will allow identification of targets for clinical intervention and treatment strategies for OSAHS-related cardiovascular diseases.

**Figure 1:**
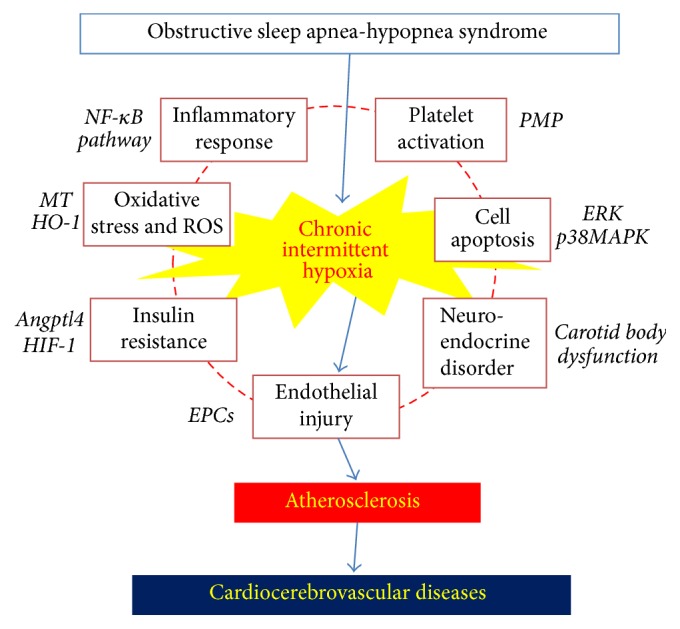
Putative mechanisms of CIH-induced atherosclerosis.

A number of researchers have investigated the correlation of risk factors, the underlying mechanism, and micromolecular levels in CIH and AS using clinical,* in vivo*, and* in vitro* studies. The underlying mechanisms of CIH that induce or promote the AS formation processes are characterized by a complex intersection and interaction (see Figure 1). Vascular endothelial injury is the initial step and an important mechanism of AS. However, in CIH-related AS, upregulation of inflammatory factors and cytokines and functional changes in inflammatory cells, local inflammation of vascular endothelium, oxidative stress injury, endothelial cell apoptosis, and general insulin resistance with subsequent systemic inflammation are all important factors for endothelial injuries. In addition to vascular endothelial injuries, inflammation and oxidative stress can induce apoptosis; islet cell apoptosis can result in IR, and IR can in turn induce inflammation that is exacerbated by neuroendocrine disorder.

Further research is being carried out to characterize the mechanism of CIH-mediated AS formation. Inflammatory and oxidative stress mechanisms have been the most studied, and NF-*κ*B is the most investigated signaling molecule. However, there are differences between the conclusions that have been drawn from* in vivo* and* in vitro* experiments, and currently there is no unified theory to explain the relationship between CIH and AS. Additional clinical research studies are required to more fully characterize this relationship.

Studies on the roles and mechanisms of CIH in the development of AS are still in their infancy. With the increasing number of OSAHS and AS patients and the further understanding of sleep-related breathing disorders, CIH-mediated AS will receive more attention from the larger public. Large-scale clinical studies and molecular level experimental research will greatly contribute to the reduction of morbidity and mortality in OSAHS-related cardiocerebrovascular diseases.

## Abbreviations

AS: Atherosclerosis
OSAHS: Obstructive sleep apnea-hypopnea syndrome
CIH: Chronic intermittent hypoxia
MI: Myocardial infarction
AHI: Apnea-hypopnea index
STEMI: ST-elevation myocardial infarction
HIF-1: Hypoxia inducible factor-1
PSG: Polysomnography
CPAP: Continuous positive airway pressure
EPO: Erythropoietin
MS: Metabolic syndrome
hs-CRP: High-sensitivity C-reactive protein
IL: Interleukin
IR: Insulin resistance
NF-*κ*B: Nuclear factor *κ*B
TNF-*α*: Tumor necrosis factor-*α*
ICAM-1: Intercellular cell adhesion molecule-1
TC: Total cholesterol
IHR: Intermittent hypoxia/reoxygenation
ROS: Reactive oxygen species
MDA: Malondialdehyde
SOD: Superoxide dismutase
MAPK: Mitogen-activated protein kinase
SREBPs: Sterol regulatory element-binding proteins
MT: Metallothionein
HO-1: Heme oxygenase-1
IR: Insulin resistance
LDL-C: Low density lipoprotein-cholesterol
SCD-1: Stearoyl-CoA desaturase-1
Angptl4: Angiopoietin-like 4
vLDL-C: Very low density lipoprotein-cholesterol
ERK: Extracellular regulatory protein kinase
VSMCs: Vascular smooth muscle cells
ox-LDL: Oxidized low density lipoprotein
EPCs: Endothelial progenitor cells
VEGF: Vascular endothelial growth factor
VCAM-1: Vascular cell adhesion molecule-1
PMPs: Platelet microparticles
cAMP-PKA: Cyclic adenosine monophosphate-protein kinase A
PI3K: Phosphatidylinositol 3-kinase.
